# Coping Strategies Associated with Emotional Adjustment during the Dyadic Experience of Infertility and Its Treatment: A Systematic Review

**DOI:** 10.34763/jmotherandchild.20242801.d-24-00013

**Published:** 2024-07-23

**Authors:** Meropi Moutzouri, Antigoni Sarantaki, George Koulierakis, Kleanthi Gourounti

**Affiliations:** Department of Midwifery, School of Health and Care Sciences, University of West Attica, Athens, Greece.; Laboratory of Epidemiology, Health Determinants and Well-Being, Division of Epidemiology, Prevention and Quality of Life, Department of Public Health Policy, School of Public Health, University of West Attica, Athens, Greece.

**Keywords:** coping behaviour, spouses, infertility, in vitro fertilisation

## Abstract

**Background:**

The aim of this systematic review was to reveal which of the coping strategies used by one partner are protective of and which pose a risk to the other partner’s psychological adjustment during the treatment of infertility.

**Material and methods:**

A systematic search of four electronic databases (PubMed, APA PsycINFO, SCOPUS, ScienceDirect), as well as the references of the retrieved articles, was performed between May and September 2023 for studies published from 1990 until 2023, using appropriate MeSH terms and associated text words. The Preferred Reporting Items for Systematic Reviews and Meta-Analyses (PRISMA) guidelines were followed. Using an a priori developed pilot data extraction form, authors performed an independent extraction of articles. Information on participants, coping mechanisms, and psychological adjustment was extracted from each study. Relevant articles were critically appraised, and a narrative synthesis was conducted based on the different designs and outcome measures among the included studies.

**Results:**

A total of 194 articles were retrieved, and 187 were excluded for not meeting the inclusion criteria. After duplicates had been removed, five studies were included in the review. The results revealed that the psychological adaptation of infertile couples at an interpersonal level may be correlated with both the type of coping and the stage of the stressor (infertility treatment or in vitro fertilisation - IVF).

**Conclusion:**

This systematic review suggests that health professionals could design and apply interventions based on modifying the coping mechanisms of infertile spouses to increase levels of well-being and decrease levels of distress.

## Introduction

Coping is an important determinant of psychological adjustment during the experience of infertility and its treatment [1, 2]. The coping strategies are behavioural and emotional efforts that are implemented in order to manage unexpected stressors and changes [3] or to regulate a situation to an individual’s desired state [4, 5]. Infertile individuals often deal with severe stressors and changes in their personal lives, couple and sexual relationships, social networks [6], financial resources and life expectations, and thus, they are likely to use coping mechanisms.

Numerous studies have used the individual as the unit of analysis to examine the coping attempts associated with emotional outcomes [2]. Nevertheless, the involvement of both partners in the experience of fertility problems and the fact that both spouses experience a variety of stressors and changes [7] provides an opportunity to extend the study of the association of coping mechanisms with emotional adjustment beyond the individual level. Coping should be viewed from a dyadic perspective, which also considers the partners’ coping strategies as both partners are involved and are affected by the experience of fertility problems or assisted reproductive technology (ART) [8]. For example, the difficulty in transition to parenthood affects both husband and wife, regardless of male or female diagnosis, since both partners cannot be parents at the present time. Moreover, both men and women experience a series of medical examinations in order to examine the fertility problem. Also, the couple has to make important decisions about dealing with infertility, such as deciding which fertility treatment to follow. In addition, although the woman usually undergoes all the medical procedures during the experience of ART, the man participates in them through support to his partner or participates actively through the production of sperm in a special area of the fertility medical unit.

Rockliff et al. [2] conducted a systematic review on the association between psychosocial factors, such as coping strategies, and psychological outcomes at the intrapersonal level. Regarding the fact that there is no review that approaches the association between coping and emotional outcome at the interpersonal level, this systematic review summarised and examined surveys exploring which coping strategies of one partner are associated with distress or well-being of the other partner during the various stages of the treatment of infertility. Thus, the aim of the review was to reveal which coping strategies are protective and which are risk factors for psychological adjustment at the dyadic level.

## Material and methods

### Eligibility criteria

There are two general categories of eligibility criteria: study characteristics and report characteristics [9, 10]. In this systematic review, studies were selected according to the criteria outlined below. Individual quantitative studies of all designs were included, whereas qualitative studies were excluded. We included only quantitative studies because these studies often provide more objective and analysable data, which are less prone to variation of interpretation; bias is usually minimised; and subjectivity is limited in their review process, ensuring a more rigorous and replicable analysis. Studies had to include husband-wife pairs of any age, women or men, experiencing infertility or its treatment (such as via IVF). Articles had to examine the association between the coping mechanisms of one partner and the psychological outcomes of the other partner. From a methodological point of view, studies had to use scales for measuring coping strategies as independent variables, and questionnaires of negative or positive psychological adjustment for measuring depression and anxiety or aspects of well-being were used as outcome variables.

### Search strategy

A systematic search of four medical and psychological electronic bibliographic databases (PubMed, APA PsycNET, SCOPUS, and ScienceDirect) was conducted between May and September 2023. The search included papers published from 1990 until 2023 and was limited to the English language. The specific search strategy was created by the author KG, who had previous experience in systematic review searching. Two authors developed and carried out the search.

A robust search strategy was developed using appropriate Medical Subject Headings (MeSH terms) and associated text words. As many synonyms as possible were included to ensure that all potentially useful articles were included. Search terms, such as “dyadic”, “couple”, “spouses”, and “partner”, were used to find surveys that relied on dyadic approach. Also, various search terms were used to cover the concept of coping mechanisms in the experience of infertility, such as “coping”, “coping behaviour”, “coping mechanisms”, and “coping strategies”. Moreover, different search terms were used in order to define the concept of infertility and its treatment, such as “infertility”, “infertile”, “infertile couples”, “in vitro fertilisation”, and “IVF”. Furthermore, additional sources were used, such as the reference sections of selected articles, to retrieve additional records that might not have been picked up by the electronic search and manual searching of relevant journals.

### Study selection

All references retrieved during the systematic research were stored in Mendeley. Two review authors independently screened the bibliography. Titles and abstracts were screened for eligibility criteria. Articles whose abstracts alluded to the search topic or were considered uncertain were selected for full-text screening. Review authors then decided whether these articles met the inclusion criteria, and if relevant, data were extracted and recorded for inclusion in this review. Systematic review data management software was not used due to the limited number of relevant reports. The level of inter-rated agreement between the two authors was high.

### Data extraction

Using a pilot data extraction form, which had been developed a priori, one review author extracted the data from included studies and then the second author checked the extracted data. Information was extracted from each included study on participants, coping mechanisms, and psychological adjustment. A summary of each publication included in this review (Table 1) and the key findings of each study (Tables 2 and 3) were extracted and recorded in preparation for data synthesis.

**Table 1.** Included studies characteristics

**Authors, date**	**Methods: study design, assessment time**	**Participants**	**Interpersonal level statistical analysis**	**Standardised measures of coping strategies**	**Standardised measures of emotional adjustment**
Berghuis & Stanton, 2002	Longitudinal, 1 week prior to alternate insemination attempt and the week after receiving a negative pregnancy result	43 couples	Hierarchical Multiple Regressions	COPE, Emotional Approach Coping scales	BDI
Kroemeke & Kubicka, 2017	Cross sectional, during ART	31 couples	APIM	Brief COPE	CES-D, PIL
Levin et al., 1997	Cross sectional, during different stages of infertility treatment	46 couples	MANOVA	CISS-SSC	BSI
Peterson et al., 2006	Cross sectional, 3 months prior to IVF	420 couples	MANCOVA	WCQ	BDI
Stanton et al., 1992	Cross sectional, during experience of infertility without IVF attempts	72 couples	Partial Correlations	WOC	GSI

**Table 2.** Studies examining the association of aspects of coping strategies of men with psychological adjustment of women during the experience of infertility and its treatment/IVF

	**Aspects of coping strategies of men**					**Outcome: Psychological adjustment of women**		
Studies	Distancing	Emotion – approach coping/Emotion – oriented coping	Substance use	Positive reinterpretation	Problem – focused coping	Pre-treatment/pre-IVF	Mid-treatment/Mid-IVF	Post-treatment (failure)/Post-IVF
Berghuis & Stanton, 2002		High use (prior to treatment)		High use	High use			Decreased depressive symptoms
Kroemeke & Kubicka, 2017			Low use				High sense of life purpose	
Peterson et al., 2006	High use					Increased depressive symptoms		

**Table 3.** Studies examining the association of aspects of coping strategies of women with psychological adjustment of men during the experience of infertility and its treatment/IVF

	**Aspects of coping strategies of women**						**Outcome: Psychological adjustment of women**		
Studies	Emotion – approach coping /Emotion – oriented coping	Self – controlling coping	Evasive coping/Avoidance	Substance use	Religious coping	Social support seeking	Pre-treatment/pre-IVF	Mid-treatment/Mid-IVF	Post-treatment (failure)/Post-IVF
Berghuis & Stanton, 2002			Low use		Low use				Decreased depressive symptoms
Kroemeke & Kubicka, 2017			High use	Low use		Low use		High sense of life purpose	
Levin et al., 1997	High use (by both women and men)							Increased distress	
Peterson et al., 2006		High use					Increased depressive symptoms		
Stanton et al., 1992		High use					Increased distress		

### Quality assessment of the reviewed articles

The methodological quality of the five included studies was evaluated by using a structured format adapted from Greenhalgh [11]. The systematic review adhered to the PRISMA guidelines [12]. Criteria for evaluating the methodological quality of included studies were: 1) Evidence of random recruitment sample; 2) response rate ≥70%; 3) evidence that each variable, such as coping strategies, distress, anxiety, depression, and well-being, was measured by using standardised, validated instruments; and 4) control of confounding variables to avoid systematic bias. Studies that provided evidence of the third criterion and met at least two out of the remaining three criteria were finally included. Quality assessment and data extraction were conducted by a single reviewer using these explicit criteria. A second reviewer then checked the quality assessment procedure.

### Data synthesis

Due to the different designs and outcome measures among the included studies, data were synthesised narratively. The key characteristics of each study, including findings, are presented in the text and in Tables 1, 2, and 3. The narrative synthesis explored the relationship and findings both within and between included studies.

## Results

### Study selection

**Figure 1.**
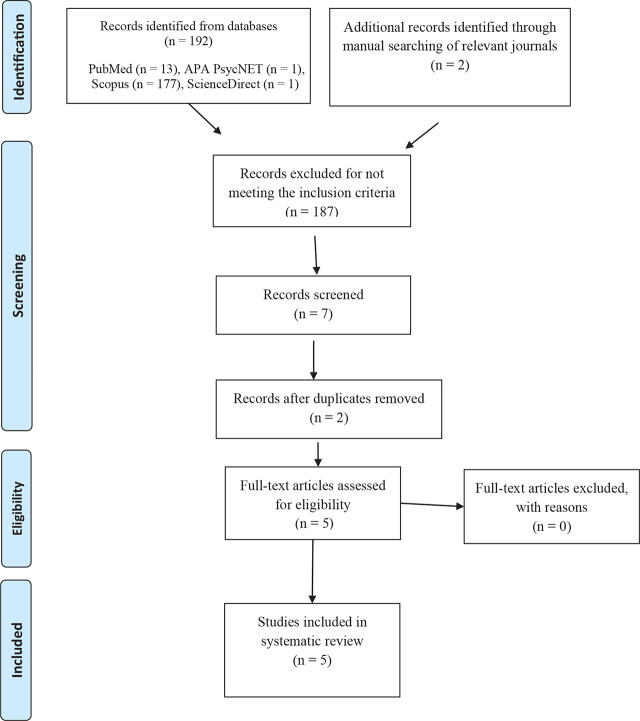
Selection process of included studies.

The initial search generated 194 titles. After the assessment of the titles, abstracts and full-texts, 187 references were excluded because they were not relevant to the search topic and did not fit the selection criteria of this review. Seven articles were selected, two of which were duplicates, leaving five studies for this systematic review. The process for identification, screening, eligibility and inclusion, which underpins this systematic search, is illustrated by the flowchart in Figure 1. The five studies included in this systematic review are described in Table 1.

## Included studies characteristics

### Study location and design

Four of the five studies took place in the United States of America [13, 14, 15, 16] and one survey in Poland [17]. Four of the included studies were cross-sectional [14, 15, 16, 17], and one survey was longitudinal [13]. Included studies used specific inclusion criteria for controlling certain confounding demographics, such as age, and medical variables, such as duration of infertility and fertility treatments received, in order to address potential sources of bias. In addition, surveys were included that incorporated statistical analyses, such as hierarchical regression, to control confounding variables.

### Participants

Samples of all reviewed studies consisted of infertile couples, but participants did not experience the same stage of infertility or its treatment. In the study of Berghuis and Stanton [13], the sample was approached one week prior to the alternate insemination attempt and the week after receiving the negative pregnancy result. In the study of Kroemeke and Kubicka [17], participants were undergoing ART. Levin, Sher and Theodos [14] included couples who were experiencing different stages of infertility treatment. In the study of Peterson, Newton and Rosen [15], the infertile dyads were approached three months prior to an IVF cycle. Stanton, Tennen and Affleck [16] included in their survey infertile couples who had not yet begun any IVF attempt.

The size of study samples ranged from 31 to 420 infertile dyads. One study included more than 100 participants [15], while four studies included fewer than 100 participants [13, 14, 16, 17]. None of the five studies had a comparison group or included two matched control groups. Both infertile men and women were participants in all five studies [13, 14, 15, 16, 17]. None of the studies in this review provided a power calculation a priori or performed a post hoc power analysis, a fact that indicated that the effect size was large and the power of the tests was good.

### Measures of coping strategies

In all included studies, coping strategies were assessed with different instruments. In the study of Berghuis and Stanton [13], the COPE and Emotional Approach Coping scales were distributed to the participants. The five COPE subscales used were Seek Social Support, Problem-Focused Coping, Avoidance, Positive Reinterpretation and Growth, and Religious Coping. Additionally, preliminary versions of the Emotional Approach Coping scales were included to assess emotional processing and emotional expression. In the study of Kroemeke and Kubicka [17], coping was assessed with the Polish version of Brief COPE. Subscales of Positive, Active Coping, Social Support Seeking, Evasive Coping and substance use were given to infertile couples. Levin et al. [14] used CISS-SSC to measure coping strategies. The CISS-SSC assesses three coping dimensions: Ask-oriented Coping, Emotion-oriented coping, and Avoidance-oriented coping. In the study of Peterson et al. [15], the WCQ was distributed to infertile couples. The instrument includes eight subscales: Escape/Avoidance, Confronting Coping, Self-controlling, Accepting Responsibility, Planful Problem Solving, Seeking Social Support, Distancing, and Positive Reappraisal. In the study of Stanton et al. [16], coping mechanisms were assessed with the WOC. Eight subscales were used: Confrontive coping, Distancing, Self-control, Seeking Social Support, Accepting Responsibility, Escape-Avoidance, Planful Problem Solving, and Positive Reappraisal.

### Measures of psychological adjustment

Psychological outcomes were conceptualised in terms of self-reported symptoms of distress, depression, and well-being. Psychological variables were assessed using generally validated tools and not infertility-specific measurements. Emotional maladjustment was assessed in all the included studies using different measurement tools, except for two that used the same instrument. More specifically, in the studies of Berghuis and Stanton [13] and Peterson et al. [15], the state of depression was measured by BDI. In the study of Kroemeke and Kubicka [17], the CES-D was used to assess the depressive mood of participants. The BSI was used by Levin et al. [14] to measure the distress of infertile couples. In the survey of Stanton et al. [16], the GSI was administered to assess the distress of infertile dyads. Nevertheless, participants’ well-being and positive adjustment, namely life purpose, during the experience of infertility and its treatment were measured by PIL in only one survey. In particular, life purpose, as a variable of well-being, was measured by PIL in only one survey [17].

### Association of coping strategies with psychological adjustment at the interpersonal level

The key findings of each study concerning the association of coping mechanisms with emotional outcomes, either maladjustment or positive psychological adaptation, at the interpersonal level are presented in Tables 2 and 3. The five included studies reported 13 significant associations. According to the depressive state of women, Peterson et al. [15] concluded that increased depressive symptoms were associated with men’s use of increased levels of distancing during the pre-treatment /pre-IVF period. On the other hand, Berghuis and Stanton [13] found that decreased depressive symptoms of infertile women after receiving a negative pregnancy result from an alternate insemination attempt were associated with men’s high use of emotional approach coping or problem-focused coping or positive reinterpretation before treatment. With regard to the positive psychological outcome of females, Kroemeke and Kubicka [17] suggested that infertile men’s low use of substances was associated with an increased sense of life purpose as an aspect of well-being during the experience of treatment/IVF.

According to the psychological maladjustment of men, Levin et al. [14] found that the increased distress of infertile men was associated with women’s high use of emotion-oriented coping during the different stages of infertility treatment. Additionally, Peterson et al. [15] concluded that the increased depressive symptoms of men during the pre-treatment period were associated with women’s high use of self-controlling coping. Moreover, Stanton et al. [16] found that the increased distress of men was associated with the high use of self-controlling coping by women during the pre-treatment period. However, Berghuis and Stanton [13] found that decreased depressive symptoms of infertile men after receiving a negative pregnancy result from an alternate insemination attempt were associated with women’s low use of avoidance or religious coping prior to treatment. With regard to the positive psychological outcomes in males, Kroemeke and Kubicka [17] suggested that infertile women’s high use of evasive coping or low use of substances or weak social support seeking were associated with men’s high sense of life purpose, as an aspect of well-being, during the treatment period.

## Discussion

The results of the current review indicate that the association of coping mechanisms with emotional outcomes at an interpersonal level depends on both the type of coping examined and the stage of the stressor (infertility treatment or IVF), such as the pre-treatment phase, mid-treatment period, and post-treatment stage.

### Pre-treatment/pre-IVF period

During the pre-treatment period, men’s increased distancing in order to deal with fertility problems was associated with women’s elevated depressive symptoms [15]. A possible explanation of this finding is that a woman may feel that her spouse does not share her worries of this demanding period, such as worries about both making important decisions to deal with infertility, and she finally feels that he is not equally committed to having children. The increased levels of depression for women may be the result of feeling unsupported by their partners.

Furthermore, before the experience of infertility and its treatment, women’s high use of self–controlling coping is related to men’s increased depressive symptoms [15] or increased distress [16]. An explanation of this result is that men’s high use of self-controlling coping efforts may reflect the more common and traditional behaviour in which men are less likely to share their difficulties and worries. As a consequence, this behaviour seems to be more acceptable and normal to the couple and may be less likely to be associated with a negative emotional adjustment of men.

### Mid-treatment/mid-IVF period

Levin et al. [14] found that high use of emotion-oriented coping by both partners was related to increased distress of men during different stages of infertility treatment. Given that treatment of infertility is characterised by numerous demands, such as financial commitment, invasive procedures, and uncertain outcomes [18], men may deal with increased distress when both partners are not actively focused on ways to manage the situation and deal with these demands. However, Kroemeke and Kubicka [17] concluded that some maladaptive coping strategies of one partner, such as evasive/avoidant coping and not social support seeking, were associated with a high sense of purpose in another partner, especially in men, during ART. An explanation of this result is that hiding worries from the spouse in order to protect him/her and avoiding open communication, difficult topics, or emotions may be the reasons why the spouse feels unburdened and has a high sense of life purpose.

### Post-treatment/post-IVF (failure) period

Berghuis and Stanton [13] found a strong between-dyad association with coping and the decrease of depressive symptoms in infertile couples who received a negative pregnancy result after an insemination attempt. First of all, men’s use of positive reinterpretation or problem-focused coping was associated with decreased depressive symptoms in women. A possible explanation is the fact that a husband’s use of positive coping strategies compensates for his wife’s lack of coping, which, in turn, seems to help reduce her depressive symptoms. Another explanation may be that women appear to benefit from their partners’ problem-focused coping attempts, perhaps because the men’s active problem-solving signals their investment in the process to their partners.

Moreover, Berghuis and Stanton [13] concluded that men’s use of emotional approach coping was related to decreased depression in women. An explanation may be the fact that emotion-focused coping is more suited to managing the outcomes of uncontrollable situations [19], such as a negative test result. This finding suggests the importance of managing emotions when stressors are considered uncontrollable. Another explanation is that husbands’ use of the emotional approach indicates their commitment to the process of treatment and test results for their spouses.

However, the five included studies did not show coherence in terms of their methodology. These differences make comparisons a difficult process. Regarding their methodology, researchers did not use the same methodological tool for estimating coping strategies or the same questionnaire in order to measure emotional outcomes. Additionally, some studies used only measurements of psychological maladjustment, such as depression and distress, while others used instruments of negative and positive adjustment, such as purpose in life. A methodological difference that refers to statistical analyses at the interpersonal level is that the five studies use different types of statistical analyses and tools in order to describe the dyadic associations.

### Limitations

The findings of this review should be interpreted after considering some methodological limitations. First, this review included only quantitative studies and articles in English. Studies that used a qualitative or mixed methodology design were excluded and, thus, a deeper understanding of the effect of coping mechanisms on psychological outcomes might be restricted to some extent. In addition, studies that were written in a language other than English were excluded, and this may have limited the range of this systematic review.

## Conclusions

### Implications for practice

According to included studies in this systematic review, some coping strategies of one partner are associated with psychological adjustment or maladjustment of the other partner at all periods of fertility treatment. The next step is to translate this knowledge into effective interventions. During counselling for infertility, systemic and dyadic approaches could help husbands and wives to successfully adjust to this demanding experience [20]. The midwives and IVF nurse practitioners who are counselling the couple regarding their infertility must be familiar with psychosocial factors, such as coping strategies, to assist in the identification of couples at high risk of psychological distress. Also, psychologists can address emotional burdens, design and apply interventions based on modifying women’s and men’s coping efforts in order to reduce their distress and the discomfort of their spouses as well as to increase their well-being in interpersonal level during the various stages of infertility and IVF cycle.

### Implications for research

Research that refers to the association of coping with emotional adjustment at the interpersonal level during the experience of infertility and its treatment is too limited. More relevant studies need to be conducted. Moreover, research can benefit from the usage of the same assessment tools of coping strategies, as well as more infertility-specific and homogenous scales of psychological outcomes. Furthermore, research needs to be periodically conducted at a specific time and at distinct stages of the experience of involuntary childlessness and its treatment. This would facilitate the comparison of results and the examination of the effect of time/stage on the association between coping attempts and psychological adaptation at the interpersonal level. Finally, future studies could benefit from the implementation of the same dyadic statistical tool in order to analyse and compare their results.

### Bulleted key points

1: During the experience of infertility and its treatment, infertile individuals use coping mechanisms associated with their emotional outcome and their partner’s psychological adjustment.
2: The association of coping mechanisms with emotional outcomes at the interpersonal level is dependent on both the type of coping examined and the stage of the stressor.
3: Interventions based on modifying coping efforts could reduce partners’ distress and their spouses’ discomfort and increase well-being at the interpersonal level.
